# Minority odors get equal say

**DOI:** 10.7554/eLife.18037

**Published:** 2016-06-23

**Authors:** Priyanka Gupta, Upinder S Bhalla

**Affiliations:** 1 National Centre for Biological Sciences, Tata Institute of Fundamental Research, Bangalore, India; 2 Cold Spring Harbor Laboratory, Cold Spring Harbor, United States; 3 National Centre for Biological Sciences, Tata Institute of Fundamental Research, Bangalore, Indiabhalla@ncbs.res.in

**Keywords:** olfaction, olfactory bulb, signal normalization, excitation/inhibition balance, Mouse

## Abstract

The olfactory system becomes more sensitive when odor inputs are weak, and less sensitive when confronted with strong odors.

**Related research article** Roland B, Jordan R, Sosulski DL, Diodato A, Fukunaga I, Wickersham I, Franks KM, Schaefer AT, Fleischmann A. 2016. Massive normalization of olfactory bulb output in mice with a 'monoclonal nose'. *eLife* **5**:e16335. doi: 10.7554/eLife.16335

**Image** A slice through the olfactory bulb, which processes information about odors. Scale bar: 100 microns.

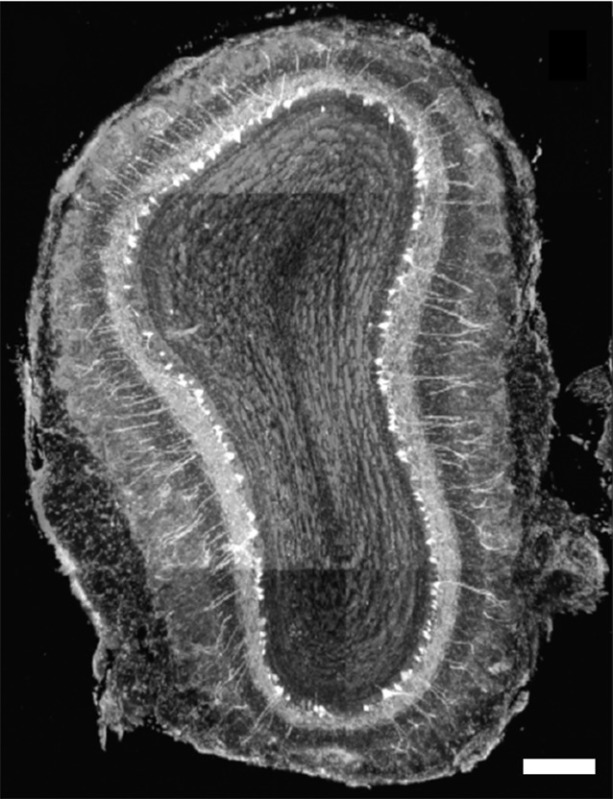

How well could you see a small green leaf if your world was enveloped in a bright purple haze? You would probably struggle to see it at all. But what if instead of detecting colors, you were detecting smells? In this case, you might detect the equivalent of the leaf pretty clearly, but hardly even notice the equivalent of the purple haze. Now, in eLife, Alexander Fleischman and colleagues – including Benjamin Roland, Rebecca Jordan and Dara Sosulski as joint first authors – report on the neural mechanisms that allow information about ‘minority’ odors to be detected through the overwhelming haze of another smell (Roland et al., 2016).

Scents are detected when odor molecules bind to odorant receptors on the surface of olfactory receptor neurons. These neurons then send signals to other neurons in a structure called the olfactory bulb, which processes this information and sends it to other olfactory regions in the brain.

To investigate the neural activity that underlies odor detection, Roland et al. – who are based at a number of institutions in France, Germany, the UK and the US – have revisited the ‘monoclonal nose’ mouse, an intriguing mouse model that was first reported in 2008 (Fleischmann et al., 2008). Mice normally express about 1100 subtypes of odorant receptor and scatter these evenly over several million receptor neurons (with each receptor neuron having one odorant receptor subtype). However, monoclonal nose mice express the same receptor (called M71) in 95% of their receptor neurons (Figure 1).

**Figure 1.**
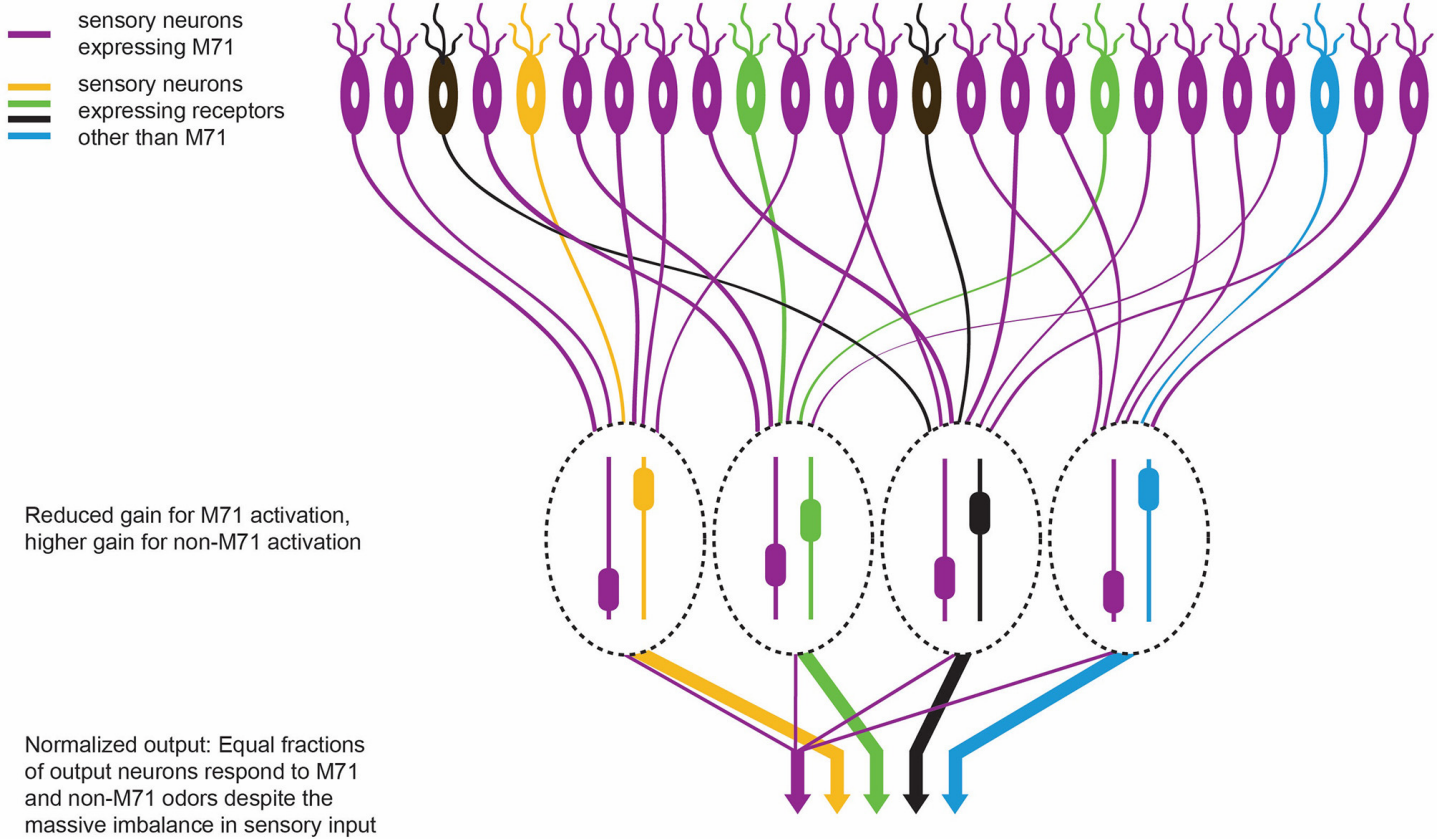
The olfactory response in M71 transgenic mice. M71 transgenic mice are genetically modified to over-express the M71 receptor (shown in purple) in olfactory receptor neurons (top). However, mice can still detect other ‘minority’ odors because the neurons in the olfactory bulb (which receive signals from the olfactory receptor neurons) become less sensitive to signals from the dominant M71 receptors, and more sensitive to signals from other receptors (shown here in yellow, green, black and blue). The heights of the sliders in the ovals represent the level of gain in that circuit: high gain leads to high sensitivity. This ultimately enables each odor to produce an equal-sized output from the olfactory bulb (represented by the width of the arrows at the bottom of the figure).

One of the more unexpected findings of the 2008 study was the apparent inability of these mice to detect acetophenone: this was surprising because acetophenone (which is found in mouse urine) is the primary molecule that activates the M71 receptor. The study involved a behavioral task in which the animals had to discriminate unscented air from air that contained various concentrations of odors. The M71 transgenic mice were capable of quite subtle discriminations for all the tested odors except acetophenone.

Roland et al. now confirm the above behavior but also show that M71 transgenic mice start sniffing when exposed to acetophenone, suggesting that they can in fact detect this odor. If the earlier results were unexpected, the new data are even more intriguing and open up possibilities for teasing apart the mechanisms that underlie these apparently contradictory behaviors.

As a starting point, Roland et al. used a wide range of measures to examine what goes on at the output of the olfactory bulb in the M71 transgenic mice. For example, they used multiphoton imaging to record the activity of populations of neurons called mitral cells that transmit information from the bulb to the other olfactory regions of the brain. They also used whole-cell patch-clamp recordings to acquire detailed readouts from individual mitral cells in both anesthetized and awake mice. These measurements showed that the responses of the mitral cells in M71 transgenic mice are very similar to those of normal mice. The fraction of cells that respond to odors, including acetophenone, was indistinguishable from that seen in normal mice. The same was true for the time courses of the responses, and also for the dependence of odor-driven mitral cell activity on the mouse’s breathing patterns. The only apparent difference was that the output responses were more variable in the transgenic mice, and were slightly weaker to non-acetophenone odors.

This is rather impressive from a computational perspective. Not only does the olfactory system amplify responses to weak odorant inputs (that is, odors other than acetophenone) in the M71 transgenic mice, it also suppresses the massive sensory input (from acetophenone) that drives 95% of the sensory neurons (Figure 1). This normalization preserves much of the information about the detected odors despite the sensory input being hugely distorted.

How does the olfactory bulb accomplish this feat? Roland et al. trace the mechanism to a change in the balance of the inhibitory inputs to the mitral cells. These inputs increase in response to acetophenone, while there is less inhibition of other ‘minority’ odors than there is in normal mice. Based on the results of optical imaging, Roland et al. ascribe this change in the pattern of inhibition to cells called periglomerular cells that are found in the input layer of the olfactory bulb, but other explanations are also possible (Mori et al., 1999; Boyd et al., 2012; Kato et al., 2013; Miyamichi et al., 2013; Banerjee et al., 2015). Future work can build on the results of Roland et al. and isolate the contributions of the various olfactory circuit elements using modern genetic approaches and the large battery of tools that neuroscientists can use to manipulate neuronal activity.

One peculiarity that remains to be resolved is the discrepancy between the neuronal and behavioral responses of M71 transgenic mice to acetophenone. However, the study by Roland et al. sets up a powerful system for understanding the computations that occur in the first stages of the olfactory system to allow different smells to be detected.

In the language of electronic circuit theory, the olfactory bulb employs a “gain-control” layer early in processing to suppress large swings in the size of the input signals it receives. The outcome of such filtering (as seen in M71 transgenic mice) is exactly what one sees in an electronic circuit forced to find a small signal on a huge background. The circuit and bulb both become a little less sensitive and a little noisier. However, a typical electronic circuit has to do this for just one signal. The remarkable accomplishment of the olfactory bulb, as highlighted by Roland, Jordan, Sosulski, Fleischman and colleagues, is to do this for a variety of different signals.
